# From mother to infant: a case report exploring postpartum depression through a cross-cultural lens in Qatar

**DOI:** 10.3389/fpsyt.2026.1763489

**Published:** 2026-03-02

**Authors:** Nada A. Al-Mulla

**Affiliations:** Department of Women & Children’s Health, Faculty of Life Sciences and Medicine, King’s College London, London, United Kingdom

**Keywords:** depression, evidence-based practice, infant, maternal health, postpartum, pregnancy, transcultural psychiatry

## Abstract

Postpartum depression (PPD) is a common psychiatric disorder affecting approximately 13% of mothers in high-income countries, with higher prevalence reported in Qatar. Its impact extends beyond maternal health, influencing paternal wellbeing and infant development. Cultural and religious factors often shape symptom recognition, care-seeking behavior, and treatment decisions, yet these influences remain underexplored in clinical practice. This report synthesizes current literature on selected maternal, paternal, and infant outcomes of PPD, with emphasis on cultural determinants of diagnosis and treatment. A case study is presented to illustrate how cultural and religious frameworks interact with biomedical models in shaping clinical care. Evidence from psychiatry, psychology, and neuroscience is integrated to highlight multidisciplinary approaches. Pharmacological interventions such as selective serotonin reuptake inhibitors (SSRIs) remain first-line, but hesitancy persists due to breastfeeding concerns and stigma. Psychotherapeutic and lifestyle interventions, including cognitive behavioral therapy, interpersonal therapy, mindfulness, and exercise, demonstrate efficacy. Faith-based practices, such as Qur’anic recitation and prayer, provide culturally meaningful coping strategies, supported by neurobiological evidence linking religiosity to emotional regulation. Paternal depression frequently co-occurs with maternal PPD, compounding risks for infant attachment and long-term socio-emotional development. PPD requires culturally sensitive, family-centered interventions that integrate biomedical and faith-based approaches. Case-based insights from Qatar underscore the importance of personalized care that respects patient beliefs while maintaining evidence-based standards. Addressing PPD holistically can mitigate intergenerational risks and promote resilience across families.

## 1 Introduction

### 1.1 Context and definition of postpartum depression

**Figure 1:**
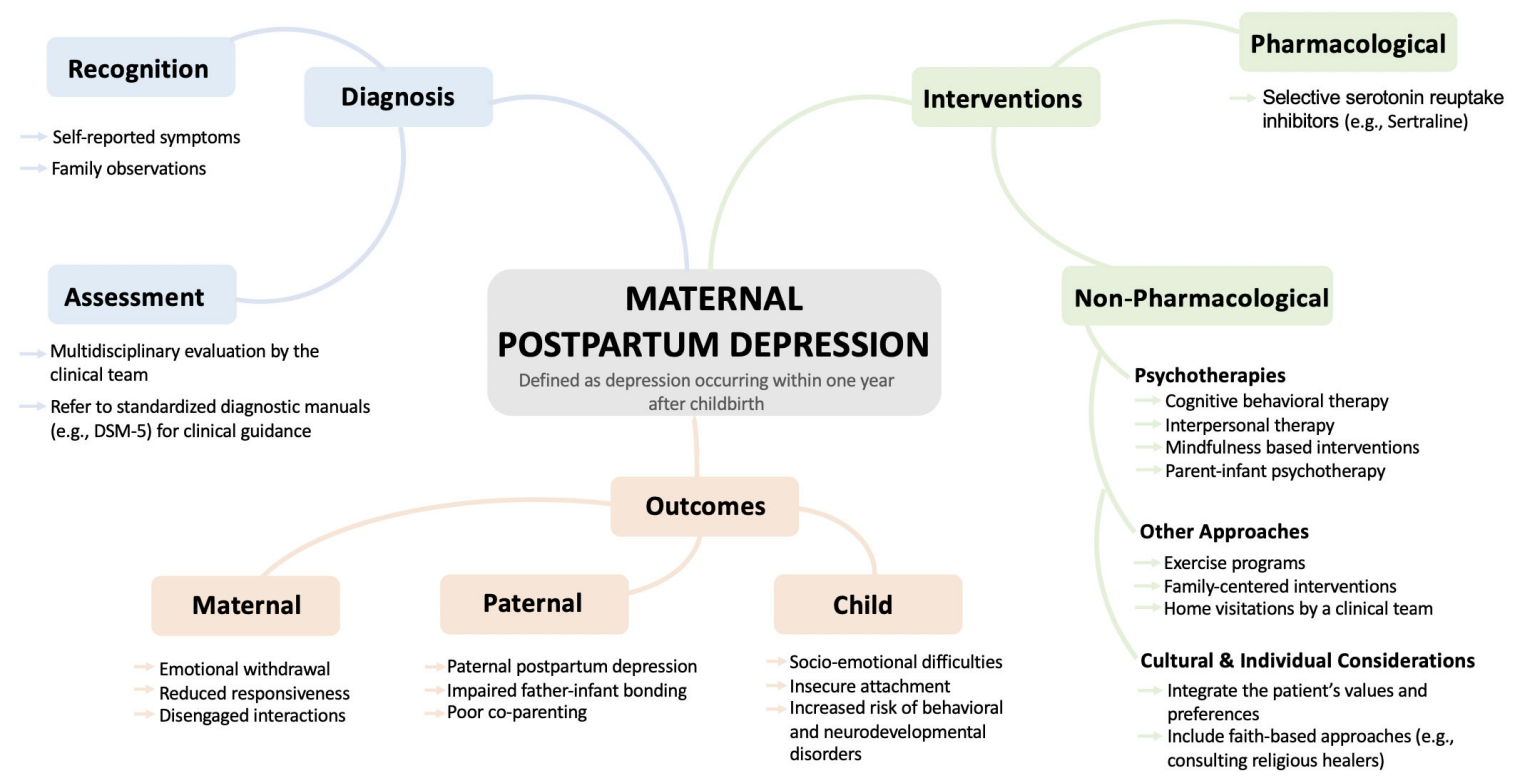
Mind map illustrating the diagnostic pathway, interventions, and selected outcomes associated with maternal postpartum depression.

Postpartum depression (PPD) is a psychiatric disorder affecting about 13% of individuals in high income countries (1). Its impact extends beyond mothers, influencing paternal mental health and infant development (2). The Diagnostic and Statistical Manual of Mental Disorders Fifth Edition (DSM-5) defines PPD as major depressive disorder with peripartum onset, occurring during pregnancy or within four weeks postpartum. For this paper, PPD refers to depression onset occurring within one year after childbirth. This report explores selected maternal, paternal, and infant health outcomes alongside cultural influences on diagnosis and treatment (see **Figure 1**), drawing on literature and the author’s clinical and cultural insights.

### 1.2 Case summary: Mrs. A’s clinical presentation

To ground this discussion in practice, a case study is presented to illustrate the clinical presentation, case management, and treatment of postpartum depression within Qatar’s cultural context. Mrs. A’s experience highlights how cultural factors can shape both symptom expression and healthcare delivery. The scenario reflects a typical clinical presentation and has been anonymized and adapted for confidentiality and educational purposes. Mrs. A, a 31-year-old multiparous woman, was admitted to the postnatal ward following an uncomplicated pregnancy within a stable marriage. Despite these favorable circumstances, she exhibited acute irritability and disengagement from her newborn daughter, which her husband identified as uncharacteristic when compared with her previous postpartum experiences. Based on findings from a comprehensive multidisciplinary assessment including obstetric, psychiatric, psychosocial, and case management input and following the exclusion of organic causes, a diagnosis of postpartum depression was made. Clinicians participated respectfully in her case management by collaborating with the family through culturally sensitive, strengths-based communication, coordinating care across disciplines, engaging in transparent shared decision making, and providing supportive follow up that both respected parental autonomy and safeguarded the wellbeing of the child.

## 2 Discussion

### 2.1 Cultural prevalence and screening practices in Qatar

In Qatar, PPD prevalence is estimated at 18%, higher than the global average and likely influenced by biological and sociocultural factors (3). Screening often uses validated tools such as the Edinburgh Postnatal Depression Scale (EPDS) and Patient Health Questionnaire-9 (PHQ-9) (4). Although these tools show high sensitivity and specificity, cultural factors may affect symptom expression and clinical presentation. In many cultures, mental illness remains highly stigmatized, leading women and family members to underreport symptoms or avoid psychiatric labels due to fears of social ostracism and familial shame. For patients like Mrs. A, clinical diagnosis guided by DSM-5 is a more robust tool.

### 2.2 Cultural frameworks and the role of family

The DSM-5 handbook does not fully account for cultural variations in mental health presentation, as such, evaluation in Middle Eastern contexts often relies on family observations, with spouses or relatives playing a central role in identifying behavioral changes. This informal surveillance often precedes clinical judgement, and family members may act as gatekeepers to care. In Mrs. A’s case, her husband’s recognition of altered behavior prompted further assessment. Religious beliefs also shape symptom interpretation; within Islamic frameworks, distress may be attributed to spiritual causes such as possession, the evil eye, or divine testing. Although Islam does not reject psychiatric diagnosis, many communities in Qatar prefer initial consultation with religious healers, which is more culturally accepted (5). A dual explanatory model integrating biomedical and religious frameworks could enhance management and align care with patient needs.

### 2.3 Pharmacological and non-pharmacological management

Management of PPD requires a multidisciplinary approach that combines pharmacological and non-pharmacological interventions while considering cultural, religious, and psychosocial contexts. Selective serotonin reuptake inhibitors, such as sertraline, are first-line treatments due to minimal transfer into breast milk (6). Despite this evidence, many mothers remain hesitant to initiate pharmacotherapy, often due to concerns about harming their infant through breastfeeding and the stigma associated with psychiatric medication (7). In Mrs. A’s case, medications to treat her PPD conflicted with her family’s religious beliefs and fears of side effects that could affect her and the infant, so she opted for non-pharmacological, faith-based interventions.

### 2.4 Psychotherapy and lifestyle interventions

Psychotherapeutic interventions are effective alternatives or adjuncts to medication. Cognitive Behavioral Therapy and Interpersonal Therapy are evidence-based approaches that target cognitive restructuring and interpersonal stressors, respectively (8). Furthermore, Mindfulness-Based Interventions and structured exercise programs have demonstrated efficacy in alleviating depressive symptoms (9). Regular physical activity, such as walking for one hour a day on five days per week, has been associated with increased hippocampal volume compared to sedentary individuals (10). Exercise not only functions as an effective antidepressant but also provides protective benefits against various chronic medical conditions.

However, when applying these interventions clinically, it is important to recognize the available literature may not be directly generalizable to the Qatari population. Nevertheless, such evidence serves as a foundational guide, with the treating physician’s clinical expertise determining the most appropriate therapeutic approach for each individual patient. In practice, clinicians should consider adopting established interventions regardless of the populations from which the data were derived and integrate the strategy with the patient’s own values, preferences, and cultural considerations to ensure that treatment plans are evidence based.

### 2.5 Religious and spiritual approaches

Cultural and religious values in certain communities strongly guide treatment decisions. Family members and religious leaders often play crucial roles in the healing process, providing guidance and emotional support. Practices such as spiritual counseling, prayer, and Qur’anic recitation are commonly employed as coping mechanisms. Neuroscientific research indicates that religious practices, including prayer and meditation, activate the frontal lobes, regions of the brain associated with attention, planning, and emotional regulation, which can enhance cognitive control and reduce stress (11). Furthermore, while numerous studies have identified various brain regions involved in spiritual and religious behaviors, a recent study highlighted a neural circuit centered in the periaqueductal gray, an area linked to altruism and emotional resilience, as fundamental to spiritual acceptance. Collectively, these findings suggest that religiosity is deeply embedded within neurobiological processes (12). However, these may not fully capture the nuances of Islamic practices in Qatar, and grouping all religious practices under a single neurobiological framework oversimplifies the diversity of experiences.

Building on this neurobiological understanding, repeated engagement in spiritual practices such as prayer and meditation fosters neuroplastic changes in brain regions responsible for emotional regulation and stress resilience. These practices strengthen neural pathways within the prefrontal cortex and limbic system, promoting adaptive coping and reducing anxiety. Research also shows that the link between religiosity and happiness is mediated by cultural values, meaning that in societies where religion is highly valued, such as Qatar, this correlation is stronger (13). Clinically, these insights underscore the importance of integrating clients’ spiritual and religious beliefs into therapy to enhance engagement and outcomes, especially for anxiety and depression (14). In Mrs. A’s case, incorporating Qur’anic recitation into her treatment plan provided a sense of relief. She reported that these practices improved her mood, showing how faith-based coping became a culturally meaningful and neurobiologically grounded part of her recovery, aligning her values with therapeutic strategies to promote emotional healing.

### 2.6 Paternal mental health and family dynamics

Furthermore, due to the emotional turmoil that Mrs. A experienced, Mr. A reported low mood after taking on caregiving responsibilities for both his wife and their newborn. This aligns with a large-scale meta-analysis highlighting a moderate correlation between maternal and paternal depression during the perinatal period (2). Such concordance is likely driven by shared psychosocial stressors, relationship strain, and cumulative caregiving demands. In Mr. A’s case, witnessing his wife’s acute emotional changes, particularly following previously stable postpartum experiences, may have placed him at increased risk of developing depression himself. Although the direction of association remains undetermined, the evidence supports a biopsychosocial model, where maternal depression functions as a psychosocial stressor that can precipitate paternal depression. This is clinically significant as the coexistence of mental health difficulties in both parents increases the likelihood of long-term adverse effects on the child, including impaired emotional regulation, insecure attachment, and a higher risk of behavioral and mental health disorders throughout development (15, 16). These findings highlight the importance of dual-parent screening and early family-centered interventions, which improve maternal depressive symptoms, enhance paternal engagement, and strengthen overall family functioning (17).

### 2.7 Infant development and intergenerational transmission

Infants depend on their mothers not only for nourishment but also for warmth and emotional interactions, fostering the basis of secure attachment and healthy development. The intergenerational transmission of mental health disorders remains an underexplored area of research, yet it represents a significant risk shaped by both genetic predispositions and the environment where the child grows. Adverse childhood experiences, such as parental mental illness, can compromise a caregiver’s ability to provide stable and sensitive care, thereby influencing the infant’s emotional and neurobiological development particularly when depressive symptoms co-occur in both parents, as this can further compromise the couple’s relationship and overall family functioning (18). At that time, Mrs. A withdrew into silence, unable to appreciate the need for loving interactions. In her absence, an external caregiver provided care. This shift in caregiving dynamics shows how parental mental health influence the child’s development.

Longitudinal evidence indicates that children of mothers experiencing depression tend to exhibit increased amygdala volume (19), suggesting the enduring neurobiological consequences of early neglect. This neglect, often resulting from maternal symptoms such as emotional withdrawal, reduced responsiveness, and disengaged interactions, may have a more profound and lasting impact on child development than direct exposure to abuse. Supporting this, further evidence shows that maternal depression during early childhood is associated with heightened hypothalamic-pituitary-adrenal (HPA) axis activity in offspring across both childhood (20) and adolescence (21), reinforcing the link between early caregiving stress and long-term physiological dysregulation.

In cases where one or both parents experience depressive symptoms, like Mrs. A’s story, the quality of parent–infant interactions can be significantly disrupted, increasing the likelihood of insecure attachment and predisposing the child to persistent socio-emotional difficulties later in life. For example, children whose fathers have depression in the perinatal period are found to have approximately a doubling of their risk of behavioral problems in childhood, over and above maternal depression (16). Extending this evidence, longitudinal research has demonstrated that postnatal paternal depression predicts depressive symptoms in offspring at age 18 (22), highlighting the enduring impact of early paternal mental health. Collectively, these findings confirm that intergenerational transmission occurs through both impaired mother-infant and father-infant interactions, emphasizing the importance of supporting both parents in the perinatal period.

### 2.8 Preventive interventions and early support

Preventing the progression of these risks requires early, family-focused interventions that support parental mental health, enhance communication between partners, and promote positive caregiving practices, thereby fostering meaningful and constructive social interactions within the family. Primary care and pediatric services play a central role in safeguarding the child’s wellbeing during this period, as they provide routine monitoring of infant growth, development, feeding, sleep, and attachment-related behaviors, while also serving as key settings for identifying parental mental health concerns that may affect caregiving. Evidence supports interventions such as fostering healthy relationships via parent-infant psychotherapy through playful activities that enhance bonding such as infant massaging, responsive play, and video-feedback interventions that enhance parental sensitivity and self-awareness (23). These practices not only enhance bonding but also act as preventive strategies, breaking the cycle of transmission and supporting long-term mental wellbeing for both parents and children. Furthermore, an effective strategy to prevent transmission is educating parents about optimal ways to interact with infants, alongside home-based interventions delivered through multidisciplinary clinical team visitations that include child psychology input, coordinated through primary care and pediatric services to assess early socio-emotional development, support parent–infant attachment, and ensure continuity of care while helping families build supportive relationships within their home environment (24).

### 2.9 Return-to-work considerations

Return-to-work practices vary across cultures, shaped by individual expectations, family norms, and workplace policies. In certain contexts, shorter maternity leave and early workforce reintegration are practiced, whereas in others, extended leave is prioritized to support maternal and infant well-being. Decisions about a mother’s return should be individualized, considering her readiness, symptom severity, occupational demands, and social support. Evidence suggests gradual reintegration is generally recommended once functional stability is achieved (25). In Mrs. A’s case, this approach was followed after a full recovery, she resumed her role as a school teacher following 12 months of maternity leave, ensuring her return occurred only when she was ready to meet the demands of her profession.

## 3 Conclusion

In conclusion, this report highlights the importance of culturally sensitive, and evidence-based interventions in addressing postpartum depression. By integrating biomedical and faith-based models, clinicians can optimize maternal and infant wellbeing while respecting cultural values. Mrs. A’s case illustrates the necessity of individualized care that honors patient beliefs, leverages family support, and adapts multidisciplinary strategies. Ultimately, addressing PPD through a personalized, holistic approach not only mitigates maternal and paternal distress but also builds a more resilient and harmonious relationship with the infant.

## Data Availability

The original contributions presented in the study are included in the article/supplementary material. Further inquiries can be directed to the corresponding author.
